# Interactome and Gene Ontology provide congruent yet subtly different views of a eukaryotic cell

**DOI:** 10.1186/1752-0509-3-69

**Published:** 2009-07-15

**Authors:** Antonio Marco, Ignacio Marín

**Affiliations:** 1 Center for Evolutionary Functional Genomics, The Biodesign Institute, Tempe, Arizona State University, USA; 2 Instituto de Biomedicina de Valencia, Consejo Superior de Investigaciones Científicas (IBV-CSIC), Valencia, Spain

## Abstract

**Background:**

The characterization of the global functional structure of a cell is a major goal in bioinformatics and systems biology. Gene Ontology (GO) and the protein-protein interaction network offer alternative views of that structure.

**Results:**

This study presents a comparison of the global structures of the Gene Ontology and the interactome of *Saccharomyces cerevisiae*. Sensitive, unsupervised methods of clustering applied to a large fraction of the proteome led to establish a GO-interactome correlation value of +0.47 for a general dataset that contains both high and low-confidence interactions and +0.58 for a smaller, high-confidence dataset.

**Conclusion:**

The structures of the yeast cell deduced from GO and interactome are substantially congruent. However, some significant differences were also detected, which may contribute to a better understanding of cell function and also to a refinement of the current ontologies.

## Background

Gene Ontology (GO) is "a set of structured vocabularies for specific biological domains that can be used to describe gene products in any organism" [1]. GO attempts to summarize the current knowledge of the basic components that shape cell function in a given organism. However, the current GO is still limited, given that we understand only part of the functions of any cell. Moreover, our current views are biased by the concentration of research efforts on some aspects of cell metabolism and function in detriment of others. This bias is caused by most data used to assign GO terms deriving from hypothesis-driven approaches.

In the last years, large protein-protein interaction (PPI) datasets have been characterized in several organisms using non-directed, massive approaches (reviewed in references [2-4]). This accumulation of knowledge is of fundamental importance, because the set of all PPIs (known as PPI graph, PPI network or interactome) may be envisaged as a functional map of the cell [3,5,6]. The fact that most interactome data have been obtained by non-directed approaches avoids the bias just described for GO. However, PPI data have also their own significant biases and shortcomings. An intrinsic problem is unavoidable: some aspects of cell metabolism may require few or no PPIs and therefore they will not be reflected in the interactome. The second problem is that so far, even in the best analyzed species, data are still partial. In addition, some protein interactions (e. g. those that occur along brief periods of time) are difficult to detect with the current methods. Finally, there is some controversy over the quality of the PPI data generated in massive, high-throughput experiments [7-11].

GO and interactome provide alternative views of how an organism is structured and functions. It is thus logical to explore whether they are congruent. This is however problematic, because GO and PPI data are very different. On one hand, gene products may be either annotated or not with GO terms. Thus, from the point of view of each GO term, the classification is dichotomous. On the other hand, PPI data are best expressed as a graph or network of units (proteins) connected by edges (known interactions). How to compare then these two, so different, types of information? The simplest way to collate GO and interactome data is to characterize from PPI results groups of densely connected units, i. e. modules [12-15] and then to establish whether modules are statistically enriched for particular GO terms. This strategy has been followed with success by several groups [12,15-18]. Discussions currently center in the best way to define modules so they make sense from either the mathematical or the biological point of view (e. g. refs. [18-20]), but it is generally accepted that modules are often enriched for particular GO terms. This congruence between GO and PPI data has led to works in which proteins are assigned functions according to the GO annotations of their interaction partners [21-23]. Similarity in GO annotations has been also used to predict interactions among pairs of proteins [24,25].

It is very significant to point out that those results imply just local congruence, but not necessarily global similarity, between the interactome and GO structures. GO and interactome could be congruent if we focus on highly connected and well-known sets of proteins, but still be very different in their global structures. In fact, in a deep sense, it is trivial to find out that proteins in a particular module often share GO annotations, if only because many modules detected correspond to, or at least include, protein complexes, which contain units that work together in the cell. Thus, all analyses performed so far fall short of addressing the general question of whether GO and PPI data offer compatible views of an organism.

It is also clear that, to characterize the level of global similarity between GO and interactome, the analysis of modules has important methodological limitations. First, proteins excluded from modules are not analyzed, so a fully global, statistical estimation of congruence is intrinsically impossible. Second, the interactome graph structure has small world properties, meaning that many units/proteins are connected to other proteins and that the distances among all them, measured as their shortest path lengths, are very small [26,27]. These problems suggest that a novel type of approach is needed. In recent works, we described novel strategies of graph analysis and we showed their usefulness to explore the structures of different complex biological graphs, such as the interactome or protein domain graphs [15,28-30]. Our methods generate hierarchical structures, dendrograms, based on the average strength of the connections among the units of a graph, and then establish whether clusters in the dendrograms are enriched for units with particular features. These procedures open the way for a global comparison of interactome and GO. Particularly, they avoid the need of selecting modules to compare with GO. In interactome-based dendrograms, it is possible to include all proteins that we wish to analyze – without dividing them into those highly connected, included in modules, and those excluded from them – and to establish whether any cluster of proteins, no matter the number of direct interactions among its members, is enriched for GO terms. As we will show, this allows for a precise mathematical determination of the similarity between the GO-based and the interactome-based classifications.

In this study, we obtained a hierarchical representation of large fragments of the interactome of *Saccharomyces cerevisiae*. Then, we determined and quantified the global similarity between a significant part of the structures of interactome and GO in the yeast. Our results greatly enrich our knowledge of the relationships between the alternative views of the yeast cell that its gene ontology and interactome provide.

## Results

### A strategy to compare interactome and GO

*Saccharomyces cerevisiae *has by far the best characterized interactome of any eukaryote. We thus decided to focus our research on this species. Our goal was to explore the yeast data and to determine whether the hierarchical structure of the GO is reflected in the interactome. We chose a simple design, based on analyzing large parent GO terms which are subdivided into several child GO terms. The question that we wanted to solve is whether we were able to detect clusters corresponding to the child terms in a dendrogram, generated from PPI data, which included all the proteins of a parent GO term. If we were able to do so, it would mean that GO and interactome have similar structures.

**Figure 1:**
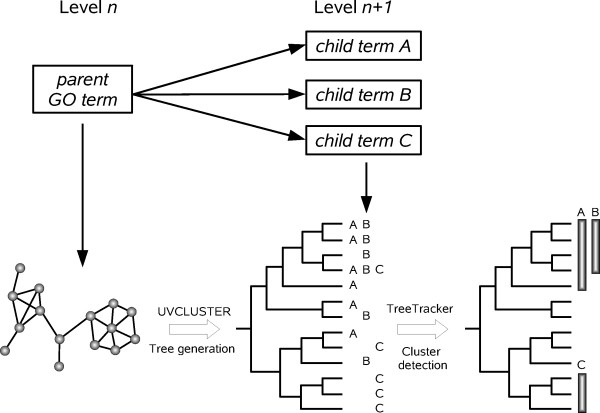
**Overview of the strategy used to compare GO and the interactome**. For a given parent GO term, we extracted the proteins annotated with it and determined their primary distances (shortest path length) in the protein interaction network. The resulting graph was transformed into a dendrogram with UVCLUSTER. We then retrieved the proteins annotated with each child GO term and labeled them in the tree. We finally detected, using the program TreeTracker, the clusters in the tree significantly enriched for each child GO term.

Therefore, our general strategy to establish the level of congruence between interactome and GO had two steps (Figure 1). First, trees were generated, using UVCLUSTER (ref. [15]; see Methods), for proteins encoded by genes included in a general, parent GO term. As indicated above, these trees are based on the relative strength of the connections among proteins, based on interactome data. Second, TreeTracker [30] was used to determine whether groups of proteins which appeared clustered together in those trees were significantly enriched for some child GO terms, hierarchically situated just below the parent term in the GO structure. If interactome and GO are congruent, we would expect to detect in a tree clusters of units enriched for the child GO terms. A significant technical point is that, because we use each parent term in isolation, we avoided the analytical problems which would derive from the fact that sometimes a GO term has several parent terms.

Table 1 summarizes the data for the nine parent terms selected for this study (see Methods for the criteria used for choosing them). Interactome data were obtained from two different databases. First, we used all the information available for *S. cerevisiae *at the Database of Interacting Proteins (DIP; ). This dataset contains both low- and high-throughput data, although about 80% of the interactions derive from massive experiments. Second, we used the "Binary gold standard dataset" (which we will call from now on "GOLD dataset"), a set of 1318 high-confidence binary interactions selected by Yu et al. [31]. The comparison between the results obtained with the DIP dataset and those obtained with the GOLD dataset will allow us to determine whether using massive data creates biases that may affect our general conclusions.

**Table 1:** Parent GO terms selected for the analysis, and number of elements included.

**GO term**	**Level^1^**	**Genes^2^**	**ORFs^3^**	**Prot**. **DIP^4^**	**Prot.DIP/** **ORFs (%)**	**Prot**. **GOLD^5^**	**Prot.GOLD/** **ORFs (%)**
Developmental process (BP)	1	768	757	632	83.5%	257	34.0%
Reproduction (BP)	1	299	298	245	82.2%	111	37.3%
Establishment of cellular localization (BP)	1	573	568	452	79.6%	188	33.1%
Response to stimulus (BP)	1	670	657	514	78.2%	207	31.5%
Ribonucleoprotein complex (CC)	2	556	459	318	69.3%	96	20.9%
Organelle envelope (CC)	2	346	345	230	66.7%	69	20.0%
Transcription regulator activity (MF)	1	307	303	276	91.1%	107	35.3%
Structural molecule activity (MF)	1	307	286	231	80.8%	75	26.2%
Transporter activity (MF)	1	380	377	297	78.8%	63	16.7%
					*Average:* *78.9%*		*Average:* *28.3%*

BP: Biological Process; CC: Cellular Component; MF: Molecular Function. ^1^: Levels of the parent GO terms. Level 1 terms are hierarchically located just below the three main categories (BP, CC and MF) while Level 2 terms are below a Level 1 term. ^2^: Number of genes selected for the analysis, i. e. those ascribed to the parent GO term which are also included in one of the selected child GO terms. ^3^: Genes among those in the previous column that contain ORFs and therefore encode for proteins. ^4^: Number of products among those in the selected ORFs for which interactions were compiled in the DIP database. ^5^: Same as ^4^, but for the GOLD dataset.

About 79% of the proteins annotated with the nine selected parent terms were included in the interactome dataset that we obtained from the DIP database. The final groups of proteins included in both the GO and the DIP interactome dataset contained from 230 to 632 units (average: 354 units; Table 1). This means that each comparison included from 4 to 11% of all *S. cerevisiae *proteins. The nine comparisons together included about 44% of the proteins present in the yeast (percentages derived from [32]; notice that a protein may be annotated with multiple terms). The GOLD dataset is much more reduced. Only 28% of the proteins annotated with one of the nine parent GO terms were found in that dataset. The average size of the groups analyzed was correspondingly much smaller than those found in DIP, including in average just 130 proteins (range 63 – 257; Table 1). In the next sections, we will first discuss the results obtained for the DIP dataset and, later, we will show that our main findings are confirmed with the smaller, high-confidence GOLD dataset.

### Interactome and GO structures are substantially congruent: DIP data

The nine selected parent GO terms were subdivided into child terms, which are detailed in Table 2. Using DIP data, we found that each child GO term included an average of 96.7 proteins. Table 2 also shows an important preliminary point, namely that interactome and GO data are largely independent. Less than 5% of the proteins analyzed in the DIP dataset were assigned to a particular GO because of PPI data in absence of other evidence (i. e. assignations annotated as "inferred from physical interaction" in GO databases). Moreover, this percentage diminishes to only 3% if two exceptional child GO terms (*Small nucleolar ribonucleoprotein complex *and *Structural constituent of cytoskeleton*) are excluded and is 0.0% for 19 of the 46 child GO terms. Therefore, we can confidently assume that, if we find evidence for global congruence between the GO and interactome structures, this will not be caused by PPI being systematically used to define to which GO terms the proteins are assigned.

**Table 2:** Summary of the GO terms used in this study.

**GO term**	**N (P)** **DIP**	**N (P)** **GOLD**	**GO term**	**N (P)** **DIP**	**N (P) GOLD**
	
**Developmental process (32502)**	**632 (16)**	**257 (8)**	**Organelle envelope (31967)**	**230 (12)**	**69 (2)**
Reproductive developmental process (3006)	26 (0)	13 (0)	Organelle inner membrane (19866)	105 (8)	27 (2)
Anatomical structure development (48856)	186 (15)	94 (8)	Organelle outer membrane (31968)	24 (0)	---
Cellular developmental process (48869)	450 (1)	169 (0)	Organelle envelope lumen (31970)	25 (0)	---
Aging (7568)	40 (0)	22 (0)	Nuclear envelope (5635)	86 (3)	35 (0)
			Mitochondrial envelope (5740)	148 (9)	34 (2)
**Reproduction (3)**	**245 (7)**	**111 (4)**			
Sexual reproduction (19953)	95 (0)	41 (0)	**Transcription regulator activity (30528)**	**276 (14)**	**107 (5)**
Asexual reproduction (19954)	74 (6)	44 (4)	Transcriptional activator activity (16563)	50 (0)	24 (0)
Reproductive process (22414)	207 (7)	88 (4)	Transcriptional repressor activity (16564)	35 (2)	13 (1)
Rep. of a single-celled organism (32505)	220 (7)	99 (4)	Transcription factor activity (3700)	45 (2)	13 (1)
			RNA polymerase II transcription factor activity (3702)	112 (4)	44 (1)
**Establishment of cellular localization (51649)**	**452 (21)**	**188 (10)**	Transcriptional elongation regulator activity (3711)	14 (6)	---
Secretion by cell (32940)	206 (9)	84 (3)	Transcription cofactor activity (3712)	36 (1)	16 (0)
Establishment of nucleus localization (40023)	17 (0)	---			
Intracellular transport (46907)	409 (21)	175 (10)	**Structural molecule activity (5198)**	**231 (29)**	**75 (18)**
			Structural constituent of ribosome (3735)	115 (0)	21 (0)
**Response to stimulus (50896)**	**514 (3)**	**207 (0)**	Structural constituent of cytoskeleton (5200)	50 (29)	31 (18)
Response to endogenous stimulus (9719)	197 (3)	101 (0)			
Cellular response to stimulus (51716)	13 (0)	---	**Transporter Activity (5215)**	**297 (8)**	**63 (1)**
Response to abiotic stimulus (9628)	83 (0)	32 (0)	Ion transport activity (15075)	111 (5)	16 (0)
Response to external stimulus (9605)	27 (0)	13 (0)	Carbohydrate transporter activity (15144)	26 (0)	---
Response to biotic stimulus (6907)	19 (0)	---	ATPase activity, coupled to movement of substances (43492)	41 (2)	---
Response to chemical stimulus (42221)	212 (0)	65 (0)	Amine transporter activity (5275)	27 (0)	---
Response to stress (6950)	370 (3)	159 (0)	Organic acid transporter activity (5342)	32 (0)	---
			Carrier activity (5386)	67 (0)	13 (0)
**Ribonucleoprotein complex (30529)**	**318 (64)**	**96 (12)**	Intracellular transporter activity (5478)	28 (0)	17 (0)
Small nuclear ribonucleoprotein complex (30532)	58 (2)	24 (0)	Protein transporter activity (8565)	48 (1)	29 (1)
Preribosome (30684)	12 (4)	---	Lipid transporter activity (5319)	11(2)	---
Spliceosome (5681)	74 (12)	33 (2)			
Small nucleolar ribonucleoprotein complex (5732)	49 (43)	10 (9)			
Ribosome (5840)	156 (5)	45 (1)			
Polysome (5844)	11 (0)	---			

Results for both the DIP and GOLD datasets are indicated. Parent GO terms are indicated in bold and, below them, the child GO terms are detailed. The numbers in parentheses adjacent to the names refer to the numerical identifiers of the GO terms. N: number of proteins for which we obtained PPI data and whose genes were annotated to the GO term. (P): in parentheses, number of proteins among those N that are annotated with the GO term based exclusively on PPI evidence. The child GO terms with less than 10 proteins found when analyzing the GOLD dataset were not further examined (dashes).

Once the data had been chosen, UVCLUSTER was used to obtain dendrograms, one per each of the nine parent GO terms (see Methods). Then, we searched for clusters of units significantly enriched for child GO terms using TreeTracker (see again the Methods section for the details). In Table 3 and Additional File 1, we describe the results obtained. Table 3 contains the summary of results for parent GO terms and Additional File 1, the details for child GO terms. We used four parameters (coverage, purity, ambiguity and Φ coefficient; see Methods for precise definitions) to quantify the results obtained. The summary of the results detailed in Table 3 is as follows: 1) Confirming that our methodology indeed detects clusters highly enriched for the corresponding GO terms, the purity of the clusters (i. e. the percentage of proteins included in a positive cluster, detected as significantly enriched for a given GO term, which indeed belong to that GO term), was high (62 – 96%, average: 80.1%). This is good evidence for our approach being very sensitive, in agreement with our previous work [30]; 2) Coverage (a measure of to which extent a given GO term is detected in the interactome data), was quite complete, ranging from 34 to 67%, with a global average of 51.2%. This means that a significant fraction of proteins in the examined GO classes are recovered in the interactome-based clusters. Interestingly, GO terms in the Biological Process category had higher coverages (average: 61.2%) than those in the Cellular Component (average: 49.7%) or Molecular Function (average: 39.0%) categories; 3) Ambiguity, which measures cluster overlap, was variable, ranging from 0 to 20% (average: 7.7%); and, 4) Finally, Phi coefficients (Φ), a precise measure of correlation between GO and interactome data (see Methods), are all positive and quite high (+0.39 to +0.64), with an average of +0.47 ± 0.03. This last result demonstrates that the GO and interactome classifications are, when globally considered, significantly similar.

**Table 3:** General results for the parent GO terms. Analyses using the DIP dataset.

**GO TERMS**	**Coverage**	**Purity** **(Average)**	**Ambiguity**	**Φ(average ± s.e.m.)**
Developmental process (32502)	63.6% (402/632)	62.2%	13.0% (74/570)	0.46 ± 0.02
Reproduction (3)	58.4% (142/245)	94.1%	0% (0/25)	0.38 ± 0.11
Establishment of cellular localization (51649)	66.8% (302/452)	88.4%	1.1% (3/264)	0.43 ± 0.10
Response to stimulus (50896)	56.4% (290/514)	77.5%	19.5% (32/164)	0.46 ± 0.05
Ribonucleoprotein complex (30529)	59.7% (190/318)	77.8%	12.8% (31/242)	0.64 ± 0.06
Organelle envelope (31967)	39.6% (91/230)	84.9%	1.2% (1/83)	0.47 ± 0.09
Transcription regulator activity (30528)	43.5% (120/276)	67.6%	15.0% (30/200)	0.40 ± 0.08
Structural molecule activity (5198)	39.8% (92/231)	95.6%	0% (0/165)	0.53
Transporter Activity (5215)	33.7% (100/297)	72.5%	6.4% (12/186)	0.43 ± 0.06

Additional File 1 details the results for all child terms. In addition of the purity, coverage and Φ coefficient values, that table also details how many significant, non-overlapping clusters were detected for each GO term and how many proteins corresponding to the GO child term were present in average in each cluster. The summary is that positive clusters were detected for 45 of the 46 child GO terms. Purities larger than 70% were observed for 31 out of those 45 child GO terms and 22 of the 46 child GO terms had coverages larger than 50%. Φ values were positive for all 45 child GO terms for which we found significant clusters. Once put aside the two already mentioned child GO terms with a high number of assignments based on PPI data, which may therefore be spuriously significant (*Small nucleolar ribonucleoprotein complex *and *Structural constituent of cytoskeleton*; see above), we determined the significance level for the other 43 child GO terms using a chi square test and Bonferroni's correction (see Methods). Φ was highly significant for 41 of those 43 terms (Additional File 1). These results further confirm that GO and interactome are notably congruent.

**Figure 2:**
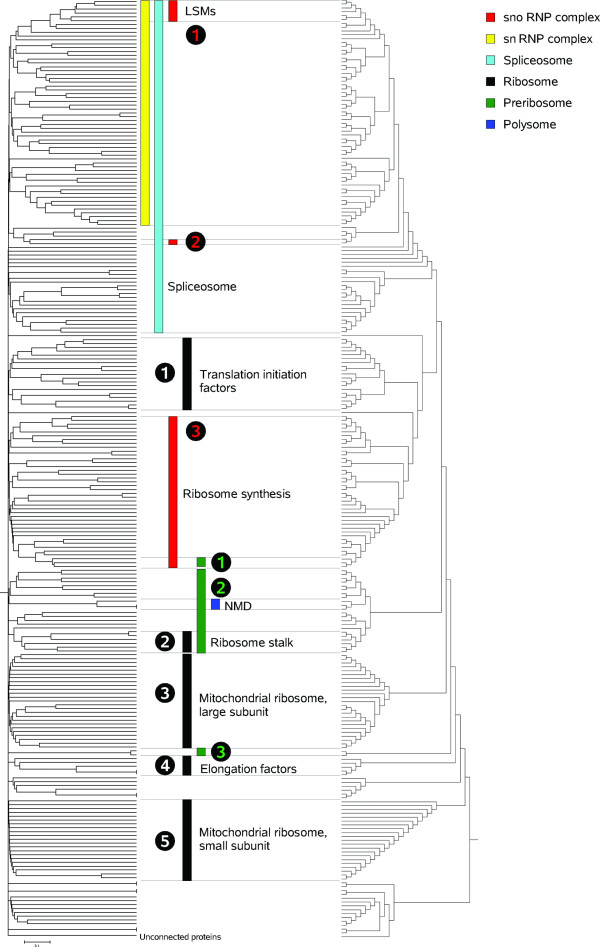
**Hierarchical representation of the protein interaction network for the *Ribonucleoprotein complex *term**. On the left, tree based on secondary distances. The tree on the right is shown to make the topology easier to visualize. At the bottom, "Unconnected proteins" are those with no direct interactions, which are separated from the rest by UVCLUSTER. Numbers refer to different clusters found for the same child GO term, which are again shown in Figure 3. snoRNP complex: Small nucleolar ribonucleoprotein complex; snRNP complex: Small nuclear ribonucleoprotein complex. NMD: nonsense-mediated mRNA decay. LSM: like-SM protein complex.

**Figure 3:**
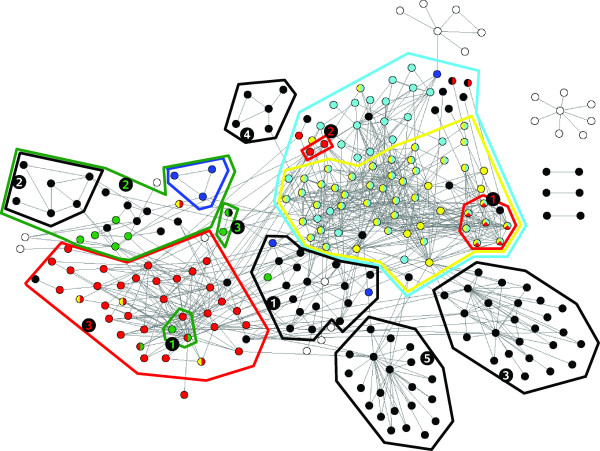
**Ribonucleoprotein complex protein interaction network**. All the proteins (dots) in this parent GO term that have at least one direct connection are shown. Colors refer to the child GO terms to which the proteins are annotated. White dots are proteins that do not belong to any of the analyzed child GO terms. The clusters detected in our analyses are framed with colored polygons. Color codes and cluster numbers as in Figure 2.

Figures 2 and 3 graphically show typical results. Figure 2 depicts the UVCLUSTER-based dendrogram of the parent GO term *Ribonucleoprotein complex*, which includes well-known cellular components such as the ribosome or the spliceosome. Significant clusters for its six child terms are indicated. Interestingly, significant clusters for four out of the six child GO terms (*Spliceosome, Ribosome, Small nucleolar ribonucleoprotein complex *and *Preribosome*) were almost completely independent, while significant clusters for the other two (*Small nuclear ribonucleoprotein complex *and *Polysome*) appeared included in more comprehensive clusters positive for other child GO terms (*Spliceosome *and *Preribosome*, respectively). This overlap explains the relatively high ambiguity of the *Ribonucleoprotein complex *term (12.8%; Table 3). In Figure 3, the graph with all the known direct PPI among the proteins in the parent GO term is shown. The color codes allow visualizing why the *Spliceosome *and *Small nuclear ribonucleoprotein complex *terms overlap in the UVCLUSTER analyses: a large number of proteins are annotated with both GO terms (shown in Figure 3 as blue/yellow dots). The high degree of purity (77.8%) for the *Ribonucleoprotein complex *GO term can be also easily visualized in this representation: notice the very few dots with a color different from that of the clusters (surrounded by the polygons). Those correspond to the few proteins included in a cluster but not annotated with the corresponding child GO term.

### Analyses of the GOLD dataset: confirming the congruence between GO and interactome

While the results shown in the previous section provide the general picture of the congruence between the GO and interactome classifications that we were interested in determining, we performed additional analyses using the GOLD dataset in order not only to validate those results, but also to check for the potential effects of low-confidence interactions in our conclusions. First, we repeated the screening for assignations to GO terms based only in PPI data, again finding that only 5.6% of the proteins included in our parent GO terms according to the GOLD dataset were in that class and that the percentage again went down to 2.7% when we excluded the same two exceptional terms *Structural constituent of cytoskeleton *and *Small nucleolar ribonucleoprotein complex*, mentioned above. Once demonstrated the almost complete independence of the GO and interactome data, we performed the same analyses that we did before for the DIP dataset. In this case, there were just 33 child GO terms containing 10 or more units. We again focused our analyses in determining whether those 33 groups appeared in the general dendrograms generated with all the proteins annotated to the parent GO terms. Table 4 shows the average results for the nine parent GO terms using the GOLD dataset. They are in general quite similar to those shown before for the DIP dataset (Table 3). As happened in the DIP analyses, both the purity (76.9%; range 64.7% – 93.6%) and coverage (average: 78.9%; range 39.3% – 96.4%) were high. Ambiguity was higher than in the DIP analyses (average 28.1%; range 0% – 46.2%). This result was however expected, considering that the number of proteins in the GOLD-based trees is much smaller than in the DIP-based trees, favoring the overlap of the significant clusters. Finally, the positive correlation between GO and interactome measured by the Φ coefficient was also highly significant and a bit higher than in the DIP-based analyses, with an average of +0.58 ± 0.06 (range: +0.37 – +0.91). This difference in average Φ coefficients for the two datasets is however statistically not significant (t test). The results for all child GO terms are detailed in Additional File 2. They were very similar to those shown before for the DIP dataset (Additional File 1). We detected significant clusters for all (n = 33) the child GO terms of size ≥ 10. Both purities above 70% and coverages larger than 50% were found in 24 of those 33 terms. After eliminating the two terms with a high assignment based solely on PPI data, we found that 29 of the 31 child GO terms left had significant Φ coefficients. All these results confirm the major findings obtained analyzing the DIP dataset.

**Table 4:** General results for the parent GO terms. Analyses using the GOLD dataset.

**GO TERMS**	**Coverage**	**Purity** **(Average)**	**Ambiguity**	**Φ(average ± s.e.m.)**
Developmental process (32502)	83.3% (214/257)	82.0%	7.2% (16/222)	0.51 ± 0.06
Reproduction (3)	96.4% (107/111)	82.5%	8.3% (1/12)	0.45 ± 0.03
Establishment of cellular localization (51649)	86.7% (163/188)	76.8%	46.2% (49/106)	0.37 ± 0.02
Response to stimulus (50896)	78.3% (162/207)	73.2%	32.1% (18/56)	0.48 ± 0.07
Ribonucleoprotein complex (30529)	82.3% (79/96)	70.7%	56.2% (41/73)	0.72 ± 0.03
Organelle envelope (31967)	87.0% (60/69)	79.5%	26.5% (9/34)	0.70 ± 0.05
Transcription regulator activity (30528)	39.3% (42/107)	64.7%	33.8% (26/77)	0.42 ± 0.03
Structural molecule activity (5198)	69.3% (52/75)	68.8%	42.3% (22/52)	0.91
Transporter Activity (5215)	87.3% (55/63)	93.6%	0.0% (0/50)	0.63 ± 0.13

### Differences between the interactome and GO structures

In spite of the clear general congruence between GO and interactome described in the previous sections, some significant structural differences were also detected in our analyses. We will base the following description mainly on results obtained from the DIP dataset, but similar considerations arose when considering the GOLD data (see some details below).

First of all, several GO terms had low coverages, meaning that PPI data to connect proteins annotated with those terms is limited or absent. The fact that PPI data is still partial obviously contributes to this problem. For example, the GO term *Ribonucleoprotein complex *had a quite high coverage (59.7% using DIP data; 82.3% using GOLD data) largely because it included several large multiprotein complexes (e. g. both units of the mitochondrial ribosome; spliceosome), for which interactome information is abundant. However, coverage could have been even higher except for the fact that PPI for proteins of the cytoplasmic ribosome were scarce. In fact, no clusters for the cytoplasmic ribosome units were detected (Figure 2). Even so, lack of PPI data does not explain all cases of low coverage. Often, proteins were annotated with particular terms by facts unrelated to them collaborating in the cell. This fact explains the especially low coverage values for some terms in the Molecular Function category, which put together proteins with related biochemical properties even if their functions are, from a biological point of view, totally unrelated. Typical in this sense were our results for the child GO term *Transcription activator activity*. In the DIP dataset, this term included 50 proteins, but only 4 proteins were detected in the UVCLUSTER dendrograms (Additional File 1). Coverage was thus one of the lowest in the whole DIP dataset, a mere 8.0%. When we searched for direct interactions among the 50 proteins annotated with this GO term, we found that just 23 loosely interacted (none of those had more than 2 interactions with other proteins in the set). It is extremely unlikely that this is solely due to PPI data for all these proteins having been missed so far. The simplest explanation is that proteins included in this GO term function alone or at most in small groups, they do not form any functional module.

**Figure 4:**
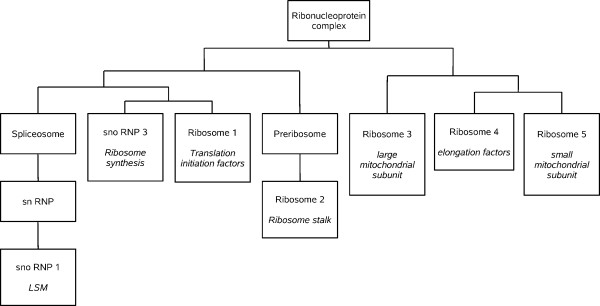
**Interactome-based structure of the GO term *Ribonucleoprotein *complex, as deduced from Figure 2**. For simplicity, significant clusters of size < 5 are omitted. This eliminates the term *Polysome*, for which only one cluster of size = 3 was found.

**Figure 5:**
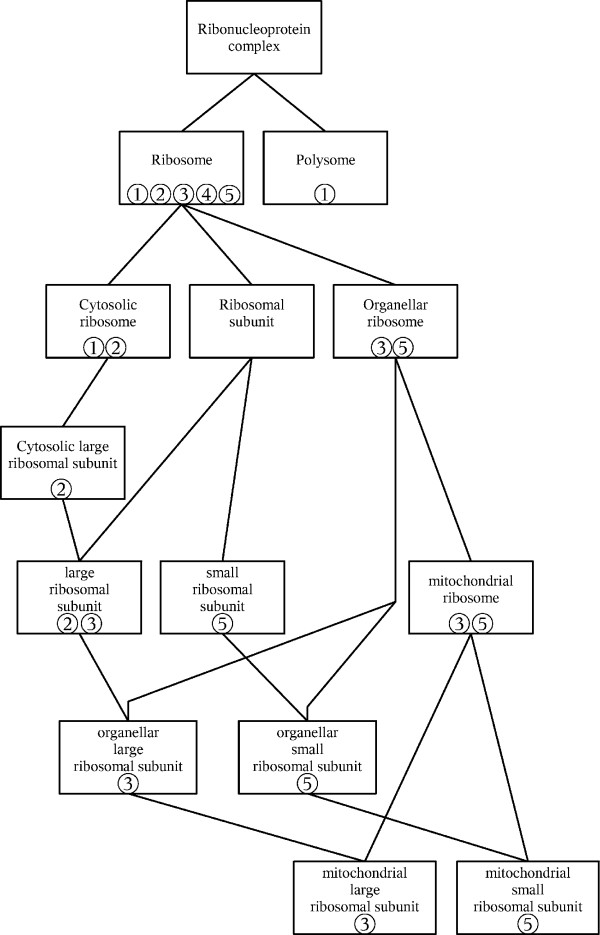
**GO terms for which it was found a significant enrichment for proteins in the clusters detected when analyzing the *Ribosome *child GO term.** Notice how this structure, directly taken from the GO, differs from that shown in Figure 4. Numbers refer to the five clusters shown also in the other figures (1: Translation initiation factors; 2: Ribosome stalk; 3: Large mitochondrial subunit; 4: Elongation factors; 5: Small mitochondrial subunit).

A second significant difference between GO and interactome structures is that most child GO terms were fragmented into multiple significant PPI clusters. For the DIP dataset, we detected in average 4.1 significant clusters for each child GO term, with 14.9 proteins per cluster (Additional File 1). Similar results were obtained for the GOLD dataset (Additional File 2). This fragmentation may be due to three different causes. First, lack of PPI data connecting the clusters, due to incompleteness of the current PPI information. Alternatively, it could be due to an artifactual division in clusters due to methodological limitations. Finally, it could also be caused by lumping of several independent cellular modules into single GO terms. Results shown in Figures 2, 3, 4 and 5 for the *Ribonucleoprotein complex *GO term, using the DIP dataset, suggest an important role for lumping (similar results were obtained for other GO terms). The GO term in those figures for which fragmentation is larger (*Ribosome*, 5 clusters) is composed by groups of proteins that belong to as many independent functional units: translation initiation factors, ribosome stalk, elongation factors and small and large mitochondrial ribosomal subunits. These functional units are largely independent according to PPI data (Figures 2 and 3). The structure deduced from the interactome is summarized in Figure 4, in which the relationships among the significant clusters of size ≥ 5 are detailed. Five of them correspond to the *Ribosome *GO term. When we then determined which GO terms among those included in the general GO term *Ribonucleoprotein complex *contained a significant number of proteins belonging to the five detected *Ribosome *clusters (see Methods), we found the results summarized in Figure 5. The fact that four clusters (nos. 1, 2, 3, 5) are detected as significantly enriched in different low-level GO terms demonstrates that the detection of multiple clusters is not spurious, but caused by real heterogeneity among the functions of the proteins included in different clusters. The appearance of multiple clusters may thus be ascribed to the fact that the general *Ribosome *GO term indeed includes independent functional units.

**Figure 6:**
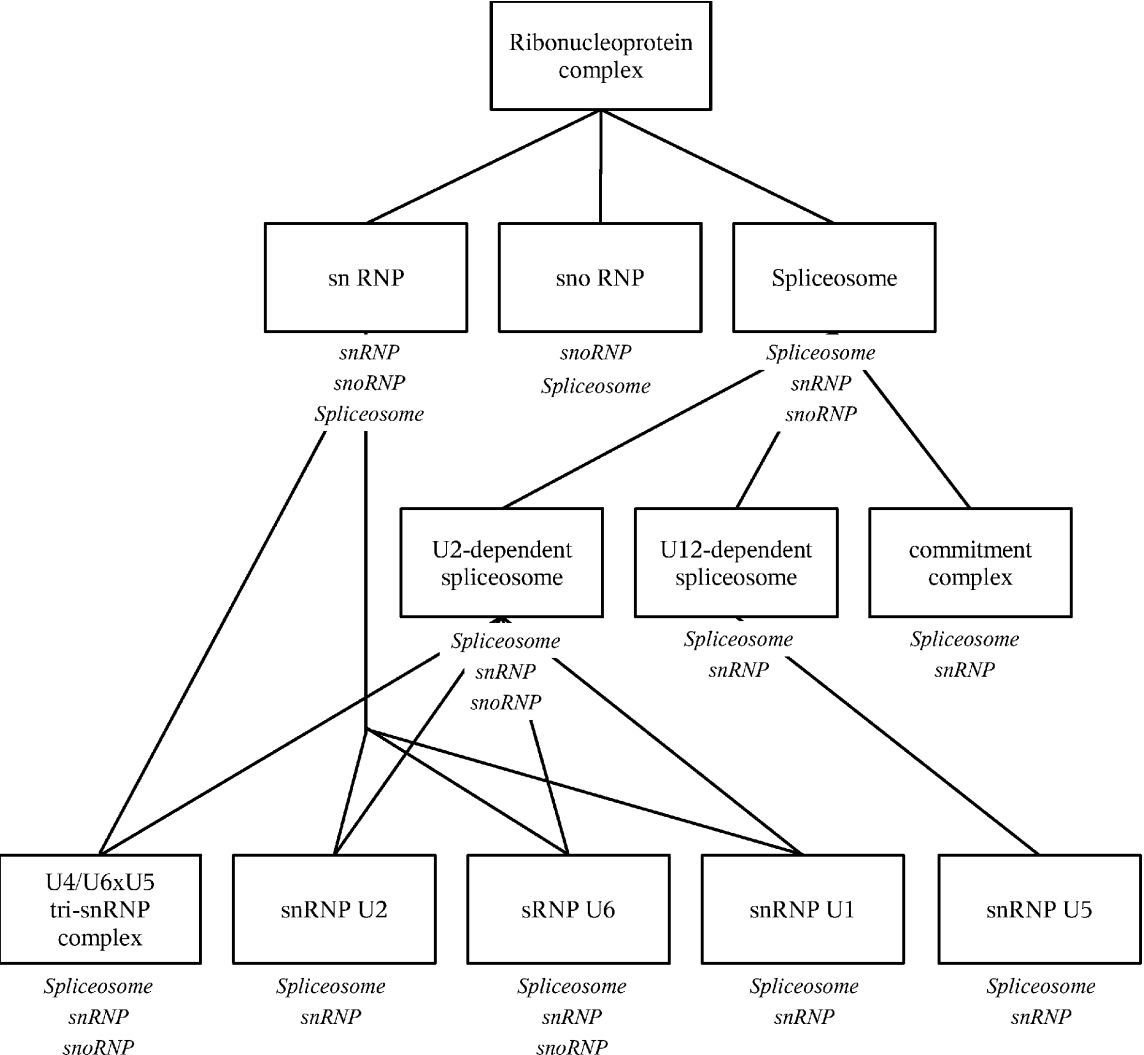
**GO terms for which a significant enrichment for proteins in the clusters detected for the child GO terms *snRNP*, *snoRNP *and *Spliceosome *was detected**. The names below the boxes refer to the child GO terms from which derive the clusters of proteins detected as significant. Notice the obvious overlap due to many proteins belonging to two or even the three child GO terms.

Figure 4 also shows the third main characteristic discrepancy that we have observed between interactome and GO: some clusters (snRNP, snoRNP 1, Ribosome 2) are included within others. This is due to multiple proteins being annotated with two or more GO terms (Figure 3). The high degree of overlapping among GO terms can be best detected when we again determine the GO terms to which the proteins in the clusters are annotated (Figures 5 and 6). In some cases (Figure 5), the degree of overlap is limited. However, in others the overlap is very considerable. For example, to generate Figure 6 we took the clusters of size ≥ 5 detected for the GO terms *Spliceosome*, *snRNP *and *snoRNP *shown in Figures 2 and 3 (a total of 4 clusters; DIP dataset) and we determined all the GO terms for which a significant enrichment of proteins in those clusters was present. Notably, all 11 GO terms detected as carrying a higher than expected number of proteins present in those clusters were actually significant for proteins included in two or even three of them (Figure 6). Similar results were found for some other GO terms.

## Discussion

In this study, we quantified for the first time the global congruence between the structures of the GO and interactome of a eukaryotic species. We used a simple scheme of analysis, which only considers large parent GO terms with multiple child GO terms. This allowed us to analyze large numbers of proteins with minimal design problems, which could be caused by using smaller groups (e. g. those lower in the GO hierarchy) or by the intrinsic structure of directed acyclic graph characteristic of the GO (which would have influenced the results in more complex designs, e. g. when using multiple GO levels). In spite of this intrinsic simplicity of design and the fact that we have not analyzed the complete GO or the whole interactome of *S. cerevisiae*, it is reasonable to expect that our results can be extrapolated to the cell as a whole. Most especially, our main conclusion, that the congruence between the structures deduced from GO and PPI is high, seems inescapable. This result goes well beyond previous efforts, which simply characterized whether groups of highly connected proteins, modules, were enriched for GO terms.

These results have important implications. A first conclusion is that our analyses show that GO classifications often have a strong structural basis: proteins annotated with the same GO term often interact, or at least they are sufficiently close in the interactome graph as to be detected in statistically significant clusters. Second, we have shown that the analyses of large PPI datasets, even those that include low-confidence interactions, provide robust results. It is true that using the GOLD dataset has led to the detection of a higher level of congruence between GO and interactome than that found using the DIP dataset (Φ coefficient for the DIP dataset: +0.47 ± 0.03; Φ coefficient for the GOLD dataset: +0.58 ± 0.06), However, this difference is statistically not significant. Therefore, the improvement obtained by excluding low-confidence interactions is scarce.

On the other hand, our results may also contribute to revise the current ontologies. For example, results in Figures 2, 3 and 4, in which we showed that the *Ribosome *term is divided into five interactome-based units, each one of them inherently logical from a functional point of view, suggest a division of this term slightly different from the one currently available. Now, only both mitochondrial subunits have their own GO terms (Figure 5). Our results suggest however that it may be better to establish terms for the five clusters detected. Another significant point to consider is why a substantial number of GO terms have low coverages. Although this can be in part explained by lack of PPI data, there are GO terms defined for groups of proteins that most likely do not interact (see results described for the *Transcription activator activity *term, above). We think that to annotate with a GO term proteins that do not work together in the cell may be acceptable for terms in the Molecular Function category, useful just for obtaining a biochemical classification of gene products. In fact, terms in that category generally had the lowest coverages (see Tables 3, 4). However, low coverages for terms in the Biological Process or Cellular Component categories should be regarded with suspicion. A careful reconsideration of these GO terms attending to the PPI data may generate a more natural classification. Finally, a third significant discrepancy between GO and interactome regards the overlaps and the hierarchical relative position of terms. The knowledge of biological networks may be very useful to define the levels in biological ontologies. One of the first goals may be to avoid as much as possible to establish at the same level two terms that contain many common proteins (e. g. Figure 6). Also, as we have seen (Figures 2 and 4), according to PPI data, a cluster for one GO term often contains a smaller cluster for another GO term of the same level. Those two terms may be based, at least in part, in just one functional module, being thus substantially redundant. This situation should be also as much as possible avoided.

## Conclusion

In summary, in *Saccharomyces cerevisiae*, GO and the global structure of the interactome show a substantial degree of congruence. This is comforting, given that both classifications have been obtained almost independently. We conclude that our current "curated" view of the yeast cell, as schematized in the GO, is globally confirmed by the unsupervised type of analysis developed here. However, the discrepancies detected mean that the current development of the *Saccharomyces *Gene Ontology is still incomplete and a better integration of PPI data may contribute to its improvement.

## Methods

We searched the GO annotations compiled in the *Saccharomyces *Genome Database (SGD; ) for large parent GO terms including 200–1000 proteins and with at least 4 child GO terms, each with 10 or more proteins. All proteins not included in a child GO term (i. e. annotated only with the parent GO term) were excluded from the cluster analyses. The UVCLUSTER program [15] (see ) was then used to obtain the hierarchical structure of the graphs for each set of proteins annotated with a GO term. The starting point to obtain the hierachical trees with UVCLUSTER analyses are the "primary distances" among the proteins (shortest path lengths in the interactome graph). They were obtained from two sources. First, from the Database of Interacting Proteins (DIP; ). We used the full *S. cerevisiae *dataset in DIP, which compiles information from multiple sources, although about 80% of the included protein-protein interactions derive from high-throughput experiments, either using the yeast two hybrid method or affinity purification of protein complexes. The second source was the "Binary gold standard set" described by Yu et al. [31], which includes only high-confidence data, mostly based on direct physical interactions characterized by the two-hybrid method. For UVCLUSTER analyses, 10000 iterations, generating as many alternative topologies, and an affinity coefficient of 100 were used to estimate the "secondary distances" that are used to build the final dendrograms (see [15] for details on these parameters). Secondary distances, obtained by weighting the 10000 alternative trees, have clear advantages over primary distances [15]. Dendrograms using secondary distances were obtained using the UPGMA routine in Mega 3 [33].

UVCLUSTER analyses are very time consuming when the number of units is higher than 1000 [15]. That is why we selected parent GO terms with at most 1000 annotated proteins. Moreover, we selected parent GO terms subdivided into multiple child GO terms to speed up the recollection and analysis of the data. We finally centered our analysis on the child GO terms containing at least 10 proteins for which interactome data were available, discarding smaller child GO terms, to avoid biases that could be caused by a few missing or a few false positive links in small groups of proteins. Some child GO terms were excluded specifically from the GOLD analyses, given that in the GOLD dataset they contained less than 10 proteins

GO is divided into three main categories: Biological Process, Cellular Component and Molecular Function. The first of these groups reflects the known information about the cellular functions in which gene products are involved, the second refers to the locations (subcellular structures, macromolecular complexes) in which those products act and the third refers to the biochemical task that the products perform (e. g. they have certain enzymatic activity, act as receptors, etc.). We retrieved four parent GO terms from the Biological Process category and three more for the Molecular Function category that comply with our criteria of selection and were hierarchically located just below these two main categories (these are often called "level 1 GO terms"). However, none of the level 1 GO terms of the Cellular Component category matched our criteria of size and number of child terms. We thus selected as parents two level 2 GO terms of that category that indeed comply with those criteria. The selected parent GO terms are summarized in Table 1.

Explorations of the dendrograms to estimate the enrichment for GO terms were performed as described in [30]. This highly sensitive method, implemented in the TreeTracker program, compares the enrichments for child GO terms in the observed tree with those in random simulations based on the same tree topology. Whenever the probability of finding by chance a particular enrichment was sufficiently low (in this study, p < 0.001; i. e. only 1/1000 of significant clusters detected are expected to be false positives) and provided that the cluster contained 2 or more units belonging to the analyzed GO term, the cluster was labeled as positive.

To quantify the congruence between GO and interactome, we used four parameters. The first one is the *coverage*, which measures to which extent a GO term is recovered by analyzing the structure of the interactome. For a parent GO term, coverage is defined as the percentage of the proteins annotated with that parent GO term that appear in the statistically significant clusters characterized for its child GO terms. For a child GO term, the definition is slightly different: coverage is defined as the percentage of proteins annotated with the child GO term that are included in significant clusters detected specifically for that term. The second parameter is the *purity *of the clusters, defined as the percentage of proteins contained in clusters significant for a given GO term which indeed are annotated with that term. The third parameter, which we called *ambiguity*, is defined as the percentage of proteins annotated with a single child GO term that however appear included in significant clusters for two or more child GO terms. Ambiguity thus indicates the degree of overlap among child GO terms according to the interactome structure. However, none of these three informative parameters (coverage, purity, ambiguity) by itself fully measures the global congruence of the two structures. To do so, we used a fourth parameter, the *Phi correlation coefficient *(Φ; [34] p. 741), defined as:



The four parameters (TP, TN, FN, FP) refer to a particular GO term. TP (true positives) are the proteins in the clusters detected as positive for a GO term which are indeed annotated to that term. TN (true negatives) are the proteins excluded from the clusters that are not annotated to the term. FN (false negatives) are proteins annotated to the GO term which are not included in any significant cluster for that term. Finally, FP are proteins included in the significant clusters that are not annotated to the GO term. Significance of Φ can be simply estimated: Φ^2 ^n, where n is the total sample size (n = FP + FN + TP + TN), follows a chi-square distribution with one degree of freedom [34-36]. Notice also that, for child GO terms, the parameters coverage and purity, explained above, can be respectively calculated as TP/(TP + FN) and TP/(TP + FP).

Finally, to generate Figures 5 and 6, we took each of the significant clusters (size ≥ 5 elements) that we wanted to analyze and we searched for GO terms that contained more proteins included in each cluster than expected by chance (p < 0.01) using High-Throughput GoMiner [37].

## Authors' contributions

Both authors devised this research. AM performed all the analyses of the paper and contributed to the text. IM wrote the manuscript. Both authors read and approved the final manuscript.

## Supplementary Material

Additional file 1 **Supplementary table 1**. Detailed results for DIP interaction network. Click here for file

Additional file 2 **Supplementary table 2**. Detailed results for GOLD interaction network. Click here for file
