# Supplementary material for: Interactome and Gene Ontology provide congruent yet subtly different views of a eukaryotic cell
Source: BMC Syst Biol. 2009 Jul 15;3:69. doi: 10.1186/1752-0509-3-69 (PMC2717056; doi:10.1186/1752-0509-3-69)
Supplement: Additional file 1 — Supplementary table 1. Detailed results for DIP interaction network. [file 1752-0509-3-69-S1.doc]

**Supplementary Table 1.** Summary of results for child GO terms, analyses using the DIP dataset. Significant φ values are in bold. *p*-values were obtained following Bonferroni’s correction (see Methods). φ values for the two terms which contained a large number of proteins whose GOs were defined according to PPI data are indicated in italics. No *p*-values were determined for those two terms.

| **GO TERMS** | **No. significant clusters**  **(No. proteins per cluster)** | **Coverage** | **Purity** | **φ**  **(*p*-value)** |
| --- | --- | --- | --- | --- |
| **Developmental process (32502)** |  |  |  |  |
| Reproductive developmental process (3006) | 3 (8.0) | 50.0 % (13/26) | 54.2 % (13/24) | **0.50 (1.1 10^-34^)** |
| Anatomical structure development (48856) | 9 (23.4) | 66.7 % (124/186) | 58.8 % (124/211) | **0.46 (9.3 10^-29^)** |
| Cellular developmental process (48869) | 18 (16.1) | 58.7 % (264/450) | 91.4 % (264/289) | **0.41 (4.3 10^-23^)** |
| Aging (7568) | 9 (6.0) | 60.0 % (24/40) | 44.4 % (24/54) | **0.48 (1.1 10^-31^)** |
| **Reproduction (3)** |  |  |  |  |
| Sexual reproduction (19953) | 4 (7.5) | 28.4 % (27/95) | 90.0 % (27/30) | **0.39 (3.4 10^-8^)** |
| Asexual reproduction (19954) | 7 (7.1) | 62.2 % (46/74) | 92.0 % (46/50) | **0.68 (6.2 10^-25^)** |
| Reproductive process (22414) | 5 (14.0) | 31.9 % (66/207) | 94.3% (66/70) | 0.17 (0.318) |
| Rep. of a single-celled organism (32505) | 5 (21.2) | 48.2 % (106/220) | 100.0 % (106/106) | **0.29 (1.8 10^-4^)** |
| **Establishment of cellular localization (51649)** |  |  |  |  |
| Secretion by cell (32940) | 14 (10.3) | 64.1 % (132/206) | 91.7 % (132/144) | **0.63 (1.3 10^-39^)** |
| Establishment of nucleus localization (40023) | 1 (4.0) | 17.7 % (3/17) | 75.0 % (3/4) | **0.35 (2.3 10^-12^)** |
| Intracellular transport (46907) | 12 (21.1) | 60.9 % (249/409) | 98.4 % (249/253) | **0.30 (3.9 10^-9^)** |

**Supplementary Table 1. (cont.)**

| **GO TERMS** | **No. significant clusters**  **(No. proteins per cluster)** | **Coverage** | **Purity** | **φ**  **(*p*-value)** |
| --- | --- | --- | --- | --- |
| **Response to stimulus (50896)** |  |  |  |  |
| Response to endogenous stimulus (9719) | 4 (53.3) | 70.6 % (137/197) | 64.3 % (137/213) | **0.45 (8.9 10^-23^)** |
| Cellular response to stimulus (51716) | 2 (2.5) | 30.8 % (4/13) | 80.0 % (4/5) | **0.49 (6.2 10^-27^)** |
| Response to abiotic stimulus (9628) | 3 (11.3) | 31.3 % (26/83) | 76.5 % (26/34) | **0.44 (1.9 10^-21^)** |
| Response to external stimulus (9605) | 5 (2.8) | 51.9 % (14/27) | 100.0 % (14/14) | **0.71 (9.1 10^-57^)** |
| Response to biotic stimulus (6907) | 3 (4.6) | 36.8 % (7/19) | 50.0 % (7/14) | **0.41 (5.5 10^-19^)** |
| Response to chemical stimulus (42221) | 7 (11.7) | 29.2 % (62/212) | 75.6 % (62/82) | **0.30 (2.3 10^-10^)** |
| Response to stress (6950) | 11 (19.7) | 56.2 % (208/370) | 95.9 % (208/217) | **0.45 (3.0 10^-23^)** |
| **Ribonucleoprotein complex (30529)** |  |  |  |  |
| Small nuclear ribonucleoprotein complex (30532) | 1 (59.0) | 84.5 % (49/58) | 83.1 % (49/59) | **0.80 (1.2 10^-44^)** |
| Preribosome (30684) | 3 (9.0) | 75.0 % (9/12) | 33.3 % (9/27) | **0.47 (1.5 10^-15^)** |
| Spliceosome (5681) | 1 (87.0) | 85.1 % (63/74) | 72.4 % (63/87) | **0.71 (1.8 10^-35^)** |
| Small nucleolar ribonucleoprotein complex (5732) | 2 (24.0) | 81.6 % (40/49) | 83.3 % (40/48) | *0.79* |
| Ribosome (5840) | 5 (15.4) | 46.8 % (73/156) | 94.8 % (73/77) | **0.52 (1.2 10^-18^)** |
| Polysome (5844) | 1 (3.0) | 27.3 % (3/11) | 100.0 % (3/3) | **0.51 (3.1 10^-13^)** |
| **Organelle envelope (31967)** |  |  |  |  |
| Organelle inner membrane (19866) | 1 (22.0) | 19.0 % (20/105) | 90.9 % (20/22) | **0.30 (3.2 10^-4^)** |
| Organelle outer membrane (31968) | 3 (5.0) | 50.0 % (12/24) | 80.0 % (12/15) | **0.60 (3.4 10^-18^)** |
| Organelle envelope lumen (31970) | 4 (4.8) | 48.0 % (12/25) | 63.2 % (12/19) | **0.50 (9.0 10^-13^)** |
| Nuclear envelope (5635) | 4 (15.3) | 67.4 % (58/86) | 95.1 % (58/61) | **0.72 (7.4 10^-26^)** |
| Mitochondrial envelope (5740) | 1 (22.0) | 14.2 % (21/148) | 95.5 % (21/22) | **0.25 (0.002)** |

**Supplementary Table 1. (cont.)**

| **GO TERMS** | **No. significant clusters**  **(No. proteins per cluster)** | **Coverage** | **Purity** | **φ**  **(*p*-value)** |
| --- | --- | --- | --- | --- |
| **Transcription regulator activity (30528)** |  |  |  |  |
| Transcriptional activator activity (16563) | 1 (4.0) | 8.0 % (4/50) | 100.0 % (4/4) | **0.26 (7.9 10^-4^)** |
| Transcriptional repressor activity (16564) | 4 (11.3) | 51.4 % (18/35) | 40.0 % (18/45) | **0.36 (7.5 10^-8^)** |
| Transcription factor activity (3700) | 1 (11.0) | 13.3 % (6/45) | 54.6 % (6/11) | **0.21 (0.020)** |
| RNA polymerase II transcription factor activity (3702) | 3 (41.3) | 69.6 % (78/112) | 62.9 % (78/124) | **0.41 (3.9 10^-10^)** |
| Transcriptional elongation regulator activity (3711) | 2 (4.5) | 64.3 % (9/14) | 100.0 % (9/9) | **0.79 (4.0 10^-38^)** |
| Transcription cofactor activity (3712) | 4 (7.3) | 38.9 % (14/36) | 48.3 % (14/29) | **0.34 (9.0 10^-6^)** |
| **Structural molecule activity (5198)** |  |  |  |  |
| Structural constituent of ribosome (3735) | 3 (17.0) | 44.4 % (51/115) | 100.0 % (51/51) | **0.53 (1.9 10^-14^)** |
| Structural constituent of cytoskeleton (5200) | 2 (22.5) | 82.0 % (41/50) | 91.1 % (41/45) | *0.84* |
| **Transporter Activity (5215)** |  |  |  |  |
| Ion transporter activity (15075) | 5 (7.4) | 31.5 % (35/111) | 94.6 % (35/37) | **0.45 (6.4 10^-13^)** |
| Carbohydrate transporter activity (15144) | 1 (4) | 11.5 % (3/26) | 75.0 % (3/4) | **0.27 (1.0 10^-4^)** |
| ATPase activity, coupled to movement of substances (43492) | 1 (13) | 29.3 % (12/41) | 92.3 % (12/13) | **0.49 (2.1 10^-15^)** |
| Amine transporter activity (5275) | 1 (34.0) | 29.6 % (8/27) | 23.5 % (8/34) | 0.18 (0.080) |
| Organic acid transporter activity (5342) | 1 (16) | 21.9 % (7/32) | 43.8 % (7/16) | **0.25 (5.3 10^-4^)** |
| Carrier activity (5386) | 2 (8.0) | 22.4 % (15/67) | 93.8 % (15/16) | **0.41 (1.1 10^-10^)** |
| Intracellular transporter activity (5478) | 1 (36.0) | 82.1 % (23/28) | 63.9 % (23/36) | **0.69 (3.6 10^-31^)** |
| Protein transporter activity (8565) | 4 (7.3) | 56.3 % (27/48) | 93.1 % (27/29) | **0.69 (9.3 10^-31^)** |
| Lipid transporter activity (5319) | 0 (-) | 0 % (0/11) | - | - |
