# Supplementary material for: Interactome and Gene Ontology provide congruent yet subtly different views of a eukaryotic cell
Source: BMC Syst Biol. 2009 Jul 15;3:69. doi: 10.1186/1752-0509-3-69 (PMC2717056; doi:10.1186/1752-0509-3-69)
Supplement: Additional file 2 — Supplementary table 2. Detailed results for GOLD interaction network. [file 1752-0509-3-69-S2.doc]

**Supplementary Table 2.** Summary of results for child GO terms, analyses using the GOLD dataset. Significant φ values are in bold. *p*-values were obtained following Bonferroni’s correction (see Methods). φ values for the two terms which contained a large number of proteins whose GOs were defined according to PPI data are indicated in italics.

| **GO TERMS** | **No. significant clusters**  **(No. proteins per cluster)** | **Coverage** | **Purity** | **φ**  **(*p*-value)** |
| --- | --- | --- | --- | --- |
| **Developmental process (32502)** |  |  |  |  |
| Reproductive developmental process (3006) | 2 (3.0) | 46.2 % (6/13) | 100 % (6/6) | **0.67 (2.1 10^-25^)** |
| Anatomical structure development (48856) | 5 (15.0) | 73.4 % (69/94) | 92.0 % (69/75) | **0.74 (7.3 10^-31^)** |
| Cellular developmental process (48869) | 2 (98.0) | 88.2 % (149/169) | 76.0 % (149/196) | **0.39 (1.6 10^-8^)** |
| Aging (7568) | 1 (5) | 13.6 % (3 /22) | 60.0 % (3/5) | **0.26 (1.0 10^-3^)** |
| **Reproduction (3)** |  |  |  |  |
| Sexual reproduction (19953) | 2 (14.5) | 46.3 % (19/41) | 65.5 % (19/29) | **0.35 (6.47 10^-3^)** |
| Asexual reproduction (19954) | 1 (48) | 79.5 % (35/44) | 72.9 % (35/48) | **0.59 (1.2 10^-8^)** |
| Reproductive process (22414) | 3 (24.0) | 76.1 % (67/88) | 93.1 % (67/72) | **0.46 (3.5 10^-5^)** |
| Rep. of a single-celled organism (32505) | 2 (35.0) | 69.7 % (69/99) | 98.6 % (69/70) | **0.39 (9.9 10^-4^)** |
| **Establishment of cellular localization (51649)** |  |  |  |  |
| Secretion by cell (32940) | 1 (149) | 97.6 % (82/84) | 55.0 % (82/149) | **0.41 (7.4 10^-7^)** |
| Intracellular transport (46907) | 4 (33.8) | 76.0 % (133/175) | 98.5 % (133/135) | **0.34 (8.6 10^-5^)** |

**Supplementary Table 2. (cont.)**

| **GO TERMS** | **No. significant clusters**  **(No. proteins per cluster)** | **Coverage** | **Purity** | **φ**  **(*p*-value)** |
| --- | --- | --- | --- | --- |
| **Response to stimulus (50896)** |  |  |  |  |
| Response to endogenous stimulus (9719) | 10 (5.0) | 49.5 % (50/101) | 100 % (50/50) | **0.58 (2.8 10^-15^)** |
| Response to abiotic stimulus (9628) | 3 (7.3) | 59.4 % (19/32) | 86.4 % (19/22) | **0.68 (6.9 10^-21^)** |
| Response to external stimulus (9605) | 1 (80) | 92.3 % (12/13) | 15.0 % (12/80) | **0.29 (1.3 10^-3^)** |
| Response to chemical stimulus (42221) | 4 (10.3) | 46.2 % (30/65) | 73.2 % (30/41) | **0.45 (3.8 10^-9^)** |
| Response to stress (6950) | 7 (31.0) | 65.4 % (104/159) | 91.2 % (104/114) | **0.38 (1.6 10^-6^)** |
| **Ribonucleoprotein complex (30529)** |  |  |  |  |
| Small nuclear ribonucleoprotein complex (30532) | 3 (6.0) | 70.8 % (17/24) | 94.4 % (17/18) | **0.77 (1.4 10^-12^)** |
| Spliceosome (5681) | 2 (10.0) | 57.6 % (19/33) | 95.0 % (19/20) | **0.65 (4.3 10^-9^)** |
| Small nucleolar ribonucleoprotein complex (5732) | 3 (17.3) | 90.0 % (9/10) | 17.3 % (9/52) | *0.25* |
| Ribosome (5840) | 1 (58) | 97.8 % (44/45) | 75.9 % (44/58) | **0.72 (6.3 10^-11^)** |
| **Organelle envelope (31967)** |  |  |  |  |
| Organelle inner membrane (19866) | 1 (44) | 100 % (27/27) | 61.3 % (27/44) | **0.60 (1.6 10^-5^)** |
| Nuclear envelope (5635) | 4 (6.5) | 74.3 % (26/35) | 100 % (26/26) | **0.77 (6.0 10^-9^)** |
| Mitochondrial envelope (5740) | 1 (44) | 100 % (34/34) | 77.3 % (34/44) | **0.74 (2.1 10^-8^)** |
|  |  |  |  |  |

**Supplementary Table 2. (cont.)**

| **GO TERMS** | **No. significant clusters**  **(No. proteins per cluster)** | **Coverage** | **Purity** | **φ**  **(*p*-value)** |
| --- | --- | --- | --- | --- |
| **Transcription regulator activity (30528)** |  |  |  |  |
| Transcriptional activator activity (16563) | 2 (4.0) | 29.2 % (7/24) | 87.5 % (7/8) | **0.44 (1.4 10^-4^)** |
| Transcriptional repressor activity (16564) | 1 (43) | 92.3 % (12/13) | 27.9 % (12/43) | **0.40 (1.3 10^-3^)** |
| Transcription factor activity (3700) | 2 (2.5) | 30.8 % (4/13) | 80.0 % (4/5) | **0.46 (6.1 10^-5^)** |
| RNA polymerase II transcription factor activity (3702) | 4 (3.8) | 34.1 % (15/44) | 100 % (15/15) | **0.48 (1.8 10^-5^)** |
| Transcription cofactor activity (3712) | 1 (43) | 75.0 % (12/16) | 27.9 % (12/43) | 0.30 (0.06, ns) |
| **Structural molecule activity (5198)** |  |  |  |  |
| Structural constituent of ribosome (3735) | 1 (24) | 100 % (21/21) | 87.5 % (21/24) | **0.91 (1.1 10^-13^)** |
| Structural constituent of cytoskeleton (5200) | 1 (62) | 100 % (31/31) | 50.0 % (31/62) | *0.38* |
| **Transporter Activity (5215)** |  |  |  |  |
| Ion transporter activity (15075) | 2 (4.0) | 50.0 % (8/16) | 100 % (8/8) | **0.65 (6.6 10^-6^)** |
| Carrier activity (5386) | 2 (2.5) | 38.5 % (5 /13) | 100 % (5/5) | **0.58 (1.5 10^-4^)** |
| Intracellular transporter activity (5478) | 2 (8.0) | 94.1 % (16/17) | 100 % (16/16) | **0.96 (8.0 10^-13^)** |
| Protein transporter activity (8565) | 1 (39) | 74.4 % (39/39) | 74.4 % (39/39) | 0.33 (0.29, ns) |
